# Genetic diversity of *Plasmodium vivax* and *Plasmodium falciparum* in Honduras

**DOI:** 10.1186/1475-2875-11-391

**Published:** 2012-11-26

**Authors:** Ana Cecilia Lopez, Andres Ortiz, Jorge Coello, Wilfredo Sosa-Ochoa, Rosa E Mejia Torres, Engels I Banegas, Irina Jovel, Gustavo A Fontecha

**Affiliations:** 1 MEIZ-Microbiology School, National Autonomous University of Honduras (UNAH), Tegucigalpa, Honduras; 2 National Malaria Laboratory, Department of National Surveillance Laboratory, Ministry of Health, Tegucigalpa, Honduras; 3 Parasitology Department, Microbiology School, National Autonomous University of Honduras (UNAH), Tegucigalpa, Honduras; 4 Malaria Research Laboratory, Infectious Diseases Unit, Department of Medicine, Karolinska University Hospital/Karolinska Institutet, Retzius väg 10, Stockholm, 171 77, Sweden

**Keywords:** *pvama*-1, *pvcsp*, *pvmsp*-1, *pfmsp*-1, *pfmsp*-2, *Plasmodium vivax*, *Plasmodium falciparum*, Honduras

## Abstract

**Background:**

Understanding the population structure of *Plasmodium* species through genetic diversity studies can assist in the design of more effective malaria control strategies, particularly in vaccine development. Central America is an area where malaria is a public health problem, but little is known about the genetic diversity of the parasite’s circulating species. This study aimed to investigate the allelic frequency and molecular diversity of five surface antigens in field isolates from Honduras.

**Methods:**

Five molecular markers were analysed to determine the genotypes of *Plasmodium vivax* and *Plasmodium falciparum* from endemic areas in Honduras. Genetic diversity of *ama*-1, *msp*-1 and *csp* was investigated for *P. vivax*, and *msp*-1 and *msp*-2 for *P. falciparum*. Allelic frequencies were calculated and sequence analysis performed.

**Results and conclusion:**

A high genetic diversity was observed within *Plasmodium* isolates from Honduras. A different number of genotypes were elucidated: 41 (n = 77) for *pvama*-1; 23 (n = 84) for *pvcsp*; and 23 (n = 35) for *pfmsp*-1. *Pvcsp* sequences showed VK210 as the only subtype present in Honduran isolates. *Pvmsp*-1 (F2) was the most polymorphic marker for *P. vivax* isolates while *pvama*-1 was least variable. All three allelic families described for *pfmsp*-1 (n = 30) block 2 (K1, MAD20, and RO33), and both allelic families described for the central domain of *pfmsp*-2 (n = 11) (3D7 and FC27) were detected. However, K1 and 3D7 allelic families were predominant. All markers were randomly distributed across the country and no geographic correlation was found. To date, this is the most complete report on molecular characterization of *P. vivax* and *P. falciparum* field isolates in Honduras with regards to genetic diversity. These results indicate that *P. vivax* and *P. falciparum* parasite populations are highly diverse in Honduras despite the low level of transmission.

## Background

Honduras reports the greater burden of malaria among all seven countries in the Central American region and has the highest proportion of *Plasmodium falciparum* cases. Despite the dramatic decrease in clinical cases occurred during the last decade, malaria remains a serious public health problem in Honduras. In 2011, a total of 7,612 cases of malaria were reported in the country, of which 92.1% were due to *Plasmodium vivax* and 7.9% were due to *P. falciparum* or mixed infections by both species (based on data from Honduran Health Ministry, 2012). Importantly, although chloroquine remains an effective drug for treatment [1], the proportion of cases of *P. falciparum* has increased from 4.1% (1,446 cases) before 2000 to the current prevalence levels.

With the exception of a few imported cases, malaria in Central America is not considered a direct cause of death but recurrent infections may produce harmful health and economic consequences on individuals, families, and communities [2]. Therefore, countries must remain vigilant and continue to implement control strategies. An effective malaria control relies upon several interventions, such as rapid diagnosis and treatment, monitoring for the emergence of drug resistance in the parasite, insecticide resistance in the mosquito vector, and vector control. However the development of an effective malaria vaccine could be the most impactful strategy for reducing the burden of malaria cases [3].

Several parasite molecules have been proposed as potential vaccine candidate antigens [4-6]. Selected antigens expressed on the parasite's surface or on the infected human blood or liver cells have greater exposure to the immune system, but this exposure is also the driving force for their greater antigenic and genetic diversity. Genetic polymorphisms in these antigens are involved in evasion of protective immune responses thus hindering the development of an effective vaccine against any *Plasmodium* species [7]. Genes encoding surface antigen proteins have been shown to be under strong evolutionary pressure [8,9]. Understanding the genetic diversity in *Plasmodium* surface antigens in parasites from different parts of the world is paramount to inform vaccine development efforts. The study of molecular diversity of malaria parasites is also important for gaining a better understanding of population dynamics and is valuable for discriminating parasite clones within infected individuals [10].

Most studies investigating the genetic diversity of *Plasmodium* have focused on *P. falciparum* surface proteins such as circumsporozoite protein (CSP) [11], merozoite surface protein 1 (MSP-1) and MSP-2 [8,10], apical membrane antigen 1 (AMA-1) [12] and glutamate-rich protein (GLURP) [13]. A similar but less extensive approach has been implemented for *P. vivax*[14,15]. The last report of *Plasmodium* genetic diversity in Honduras dates back to 1999 [16] and is limited to *P. falciparum.* Hence, the present study on the molecular characterization of malaria parasites in Honduras aimed to undertake the genetic characterization of both circulating species in the country.

## Methods

### Sample collection

**Figure 1:**
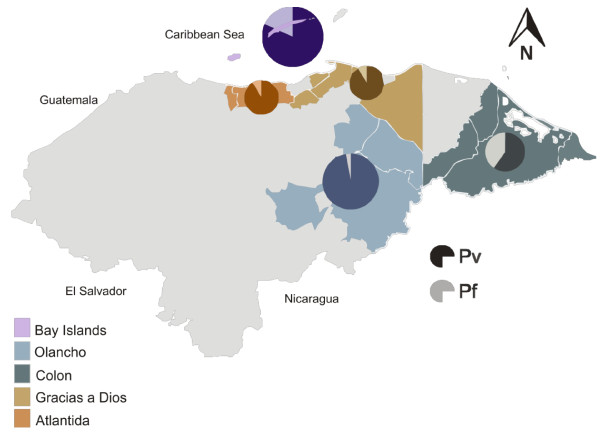
**Honduras map showing study sites by Department in five different colors.** Darker and lighter segments of the circles indicate the proportion of samples with *Plasmodium vivax* and *Plasmodium falciparum* infection respectively. Circle size shows the number of samples by Department.

Sample collection was carried out from September 2010 to September 2011 in 20 municipalities from five malaria endemic Departments of Honduras (Figure 1). Scientific approval and ethical clearance was obtained from the Ethics Board Committee of the Hearth-Lung National Institute, Ministry of Health. Informed consent was sought from all patients prior to recruitment. All patients were followed and anti-malarial treatment was readily available. Samples included in this study were chosen randomly according to positive malaria microscopy and geographic representativeness.

### Microscopy and PCR confirmation of *Plasmodium* species

Thick blood smears were stained with 3% Giemsa for 30 min at room temperature. Smears were analysed by experienced microscopists and parasite species were registered and confirmed by 18s rRNA nested PCR approach. Primer design and cycling conditions were carried out according to Singh *et al.*[17]. Genomic DNA from *Plasmodium*-positive filter paper blood spots was isolated using a Chelex-based method [18].

### Molecular markers

The *pvama*-1, *pvcsp*, *pvmsp*-1 (Block F2), and *pfmsp*-2 *loci* were amplified and sequenced in samples that had been microscopically identified as malaria cases and confirmed by polymerase chain reaction (PCR). The *pfmsp*-1 *locus* was amplified to determine allele families and allelic frequencies. Eighty-five and thirty samples diagnosed and confirmed to be infected with *P. vivax* (Pv) and *P. falciparum* (Pf) respectively were included in the study.

For *pvama*-1, DNA was amplified using primers and conditions described previously [19], with minor modifications. Briefly, primers were used at a final concentration of 0.4 μM in a 50 μl reaction mixture containing 4 μl of DNA and 2X Taq polymerase Master Mix (Promega). Primer sequences are showed in Table 1. Reaction conditions were as follows: 95°C/5 min; 35 cycles of 95°C/1 min, 56°C/1 min, 72°C/1 min. Initial denaturation and a final extension step were included for all reactions. *Pvcsp* and *pvmsp*-1 genes were amplified according to Imwong *et al.*[20] by a nested and semi-nested PCR approach, respectively. Both amplification reactions for each locus were carried out in a total volume of 50 μL and in the presence of 0.4 μM of each oligonucleotide primer and 2X Taq Master Mix (Promega). The primary PCR for *pvcsp* was performed with 10 μL of DNA with the following cycling parameters: 95°C/5 min: 25 cycles of 94°C/1 min, 58°C/2 min, 72°C/2 min; 72°C/5 min. Secondary PCR was done using 1 μl of amplification product from the primary PCR. The cycling parameters for the secondary PCR were the same as for primary, except that annealing was set at 62°C and 30 cycles. The first PCR to amplify the F2 segment of *pvmsp*-1 included the following parameters: 95°C for 5 min; 25 cycles of 94°C/1 min, 50°C/2 min, 72°C/2 min; 72°C/5 min. Secondary PCR involved the same reverse primer from the first PCR. Same cycling parameters were used in the second PCR with the exception of the annealing temperature (64°C) and the number of cycles (35).

**Table 1:** Primer sequences for PCR amplification of the five molecular markers

		
*Pvama*-1		5′–CCAGCTGGAAGATGTCCTGT–3′ (F)
		5′–TAATCCGAACTTGGCGTTTC–3′ (R)
*Pvcsp*	Primary	5′–ATGTAGATCTGTCCAAGGCCATAAA–3′ (F)
		5′–TAATTGAATAATGCTAGGACTAACAATATG–3′ (R)
	Secondary	5′–GCAGAACCAAAAAATCCACGTGAAAATAAG–3′ (F)
		5′–CCAACGGTAGCTCTAACTTTATCTAGGTAT–3′ (R)
*Pvmsp*-1 (F2)	Primary	5′–GATGGAAAGCAACCGAAGAAGGGAAT–3′ (F)
		5′–AGCTTGTACTTTCCATAGTGGTCCAG–3′ (R)
	Secondary	5′–AAAATCGAGAGCATGATCGCCACTGAGAAG–3′ (F)
		5′–AGCTTGTACTTTCCATAGTGGTCCAG–3′ (R)
*Pfmsp*-1	Primary	5′–CTAGAAGCTTTAGAAGATGCAGTATTG–3′ (F)
		5′–CTTAAATAGATTCTAATTCAAGTGGATCA–3′ (R)
	Secondary K1	5′–AAATGAAGAAGAAATTACTACAAAAGGTGC–3′ (F)
		5′-GCTTGCATCAGCTGGAGGGCTTGCACCAGA–3′ (R)
	Secondary MAD20	5′–AAATGAAGGAACAAGTGGAACAGCTGTTAC–3′ (F)
		5′–ATCTGAAGGATTTGTACGTCTTGAATTACC–3′ (R)
	Secondary RO33	5′–TAAAGGATGGAGCAAATACTCAAGTTGTTG–3′ (F)
		5′–CAAGTAATTTTGAACTCTATGTTTTAAATCAGCGTA–3′ (R)
*Pfmsp*-2	Primary	5′–GAAGGTAATTAAAACATTGTC–3′ (F)
		5′–GAGGGATGTTGCTGCTCCACAG–3′ (R)
	Secondary FC27	5′–GCTTATAATATGAGTATAAGGAGAA–3′ (F)
		5′–GCATTGCCAGAACTTGAA–3′ (R)
	Secondary 3D7	5′–GCTTATAATATGAGTATAAGGAGAA –3′ (F)
		5′–CTGAAGAGGTACTGGTAGA–3′ (R)

*Pfmsp*-2 was amplified following an adapted nested PCR method reported by Mwingira *et al.*[13]. In brief, primary PCR reaction conditions were adjusted to a final volume of 50 μL including 5 μL of DNA and cycle conditions for primary PCR were 95°C/5 min; 30 cycles of 94°C/1 min, 60°C/2 min, 72°C/2 min; and a final extension at 72°C/10 min. In order to reduce the carryover of primary PCR primers into the nested PCRs, only one μL of primary PCR product was amplified in the two nested PCR reactions with the following cycle conditions: 94°C/2 min; 30 cycles of 94°C/30 s, 50°C/45 s, 72°C/2 min; and one final step of 72°C/10 min. Two separate reactions were required to amplify one out of two possible allelic families FC27 and 3D7. Specific oligonucleotides for both families were used.

*Pfmsp*-1 *locus* was amplified according to Schoepflin *et al.*[10]. The three possible variants or allelic families: K1, MAD20 and RO33 were analysed. The second variable block of *pfmsp*-1 was amplified by a nested PCR. Primary and secondary PCRs were performed in a total volume of 50 μl containing 10 μl and 1 μl of DNA, respectively. PCR primers were used at a final concentration of 0.4 μM. Three secondary and independent PCRs were run for each allelic family using specific oligonucleotides.

All reactions were carried out in a Veriti® Thermal cycler (Applied Biosystems, USA). Amplification products were analysed by electrophoresis on a 2% agarose gel stained with ethidium bromide. Sequencing of gene fragments was carried out directly from purified PCR products on both strands with their respective inner primers following standard sequencing protocols at Macrogen Corp (USA) facilities.

Gene sequences from the three *P. vivax* markers and *pfmsp*-2 were aligned using Chromas Pro (Technelysium Pty Ltd, Australia) and Mega5 software [21], and then compared with gene sequences previously published in world databases. Alignment, comparison of nucleotide and amino acid sequences, and phylogenetic tree constructions were done by using Mega5 software. Translated amino acid sequences were aligned amongst themselves and also with sequences available in GenBank [22]. Allele and family frequencies for *pfmsp*-1 were further compared among the population and were calculated by dividing the number of samples with a certain allele by the number of samples with an identifiable allele at that position. Sequences reported in this paper have been deposited in the database [GenBank: JQ903587–JQ903613].

## Results and discussion

Malaria transmission in Honduras has decreased notoriously during the last 10–12 years, and is concentrated in six (out of 18) Provinces located along the Northeastern and Atlantic Regions [23]. Samples included in this study are representative of most common areas where malaria is present. Concerning the *Plasmodium* species circulating in Honduras, *P. vivax* causes more than 90% of malaria cases and the remaining are caused by *P. falciparum*. Accordingly, this study has included a similar proportion of samples for each parasite species.

Of 85 samples subjected to *pvama*-1 gene amplification, 77 were PCR-positive and yielded a 466 nucleotide long sequence. The failure in amplifying 8 out of 85 samples could be attributed to mutations in the primers targets or technical biases. This fragment was translated into a 151–154 amino acid sequence corresponding to the partial Domain I of the gene (positions 90 to 244).

**Figure 2:**
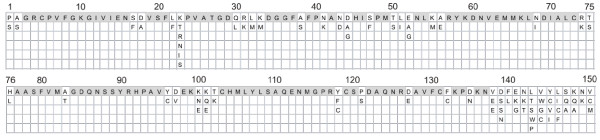
**Amino acid polymorphisms in Domain I of AMA-1 coding region of 77 *Plasmodium vivax *isolates from Honduras.** The positions of amino acid substitutions are specified vertically above. Gray boxes indicate invariable sites.

Among all *pvama*-1 sequences 41 unique amino acid haplotypes were identified with prevalence ranging from 1.38% to 27.77% confirming a high diversity in the *pvama*-1 gene among Honduran isolates. Sequence analysis revealed that 48 bp (10.3%) were polymorphic while the remaining 418 positions were conserved (Figure 2). Thirty-four haplotypes were unique, found in only one isolate each. GenBank *pvama*-1 amino acid sequences showed that 74 of 77 haplotypes revealed maximum identities from 91% to 99% compared with previous sequences reported from other regions of the world. The remaining three haplotypes showed 100% identity with *ama-1* sequences reported from other countries.

A low degree of polymorphism has been suggested for PvAMA-1 compared to other surface antigens such as PvMSP gene family [24,25]. However, more recently Thakur *et al.*[26] reported 49 different haplotypes covering both domains I and II from 61 Indian isolates (80.32% polymorphic haplotypes), and similar studies in Sri Lanka and Myanmar showed 15 haplotypes in 23 isolates (65.21%) and 34 haplotypes from 76 isolates (44.73%) [27,28]. Even though those studies included only Asian isolates, another report shows 25.71% polymorphic haplotypes within 105 isolates [29] using South American samples. The results suggest that Honduran parasites show an allelic richness more similar to Asian isolates at this locus.

**Figure 3:**
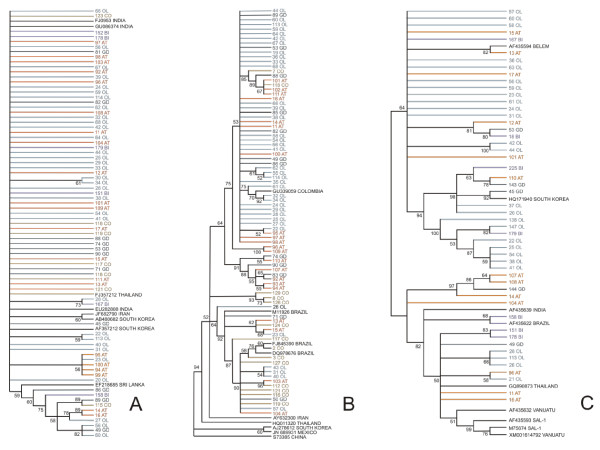
**Neighbour-joining trees with 10,000 bootstrap replicates showing relationships between: (A) *pvama*-1; (B) *pvcsp*; and (C) *pvmsp*-1 haplotypes among Honduran *Plasmodium vivax *isolates.** The evolutionary distances were computed using the Kimura 2-parameter method. Homologous sequences from other Countries were included (GenBank accession numbers are included in the dendrograms).

A phylogenetic tree depicting the relationships among *pvama*-1 haplotypes observed among Honduran parasites (Figure 3) revealed no geographic region-based clustering of the alleles. Therefore, the distribution of haplotype patterns did not differ substantially among geographic regions within the country, which is not a surprising finding, given that Honduras extends over a relatively small territory. Isolates of Asian origin (Thailand, Iran, South Korea, India and Sri lanka) were included together with Honduran *pvama*-1 sequences to determine clustering patterns but those also grouped randomly throughout the dendrogram. Similar results were found in Brazil by Grynberg *et al.*[29], who suggested that American *P. vivax* isolates seem not to derive from any particular worldwide lineage. In agreement with observations made by Putaporntip *et al.*[30], New World parasite populations show a higher geographic homogeneity compared to Old World subdivision patterns.

**Figure 4:**
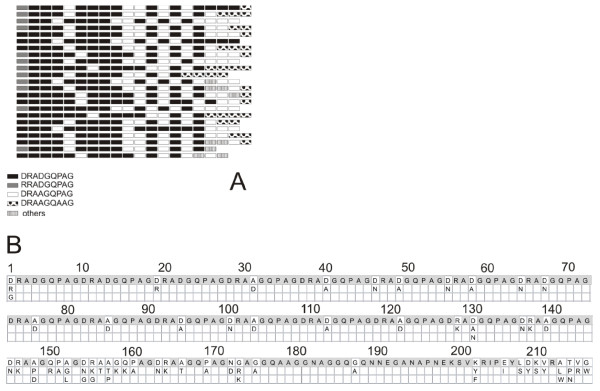
**(A) A schematic representation of the amino acid motifs of the 23 allelic *pvcsp *variants bearing the VK210 repeat type.** Each row represents a different variant. (**B**) Amino acid polymorphic positions in the *pvcsp* gene. Amino acid substitutions are specified vertically above. Gray boxes indicate invariable sites.

Nucleotide sequences from the central repeat domain of *pvcsp* were determined. A 697 bp sequence was obtained from 84 isolates. The number of single nucleotide polymorphic positions was 56 (8%). Translated nucleotide sequences revealed repetitive 9-mer motifs ranging from 17 to 20 units. The frequencies of type A (20 repeats), type B (19 repeats), type C (18 repeats) and type D (17 repeats) were 60.7%, 34.5%, 3.5% and 1.2%, respectively (Figure 4). This distribution reveals that types A and B dominate in Honduran parasites. According to amino acid composition of repeats, previous reports classified this sequence into two major types: VK210 and VK247, according to the amino acid composition of repeats [31]. In all Honduran samples the repeated motif matched almost entirely the VK210 subtype DRAA/DGPQAG with 4 residues showing changes (D/R RA A/D GQ P/A AG) compared to the original sequences described. Taking into account the arrangement of these blocks, it is possible to determine 23 different allelic types among the 84 isolates (27.38% allelic variants). None of the isolates revealed any other subtype previously reported (Figure 3). Predominance of VK210 subtype is a common finding among *P. vivax* isolates worldwide [15,32], however, according to previous studies including isolates from Mexico and South America, VK247 genotype seems to be less prevalent among New World parasites [32-34]. The results from the present study seem to support this finding because of the absence of the VK247 genotype among Honduran isolates. Since occurrence of variants others than VK210 have been previously confirmed mostly in mixed infections [34,35], it is possible that underestimation of mixed genotypes occurred in this study because of sequencing limitations.

Some authors have reported a correlation between *pvcsp* phenotypes (using a serological approach) infecting different species of *Anopheles*. Rodriguez *et al.*[36] demonstrated in the South of Mexico that *Anopheles albimanus* was more susceptible to infections by the VK210 subtype, while *Anopheles pseudopunctipennis* was more susceptible to VK247. Other publications show a different scenario where all variants of *P. vivax* were present in circulating mosquitoes of different species of South America [37,38]. Even though the characterization of vector species is out of the goals of this article, twelve species of *Anopheles* have been described in Honduras (personal communication), and the most important species are *An. albimanus*, *Anopheles darlingi*, *An. pseudopunctipennis* and *Anopheles crucians*[23]. According to those data and a only one genotype of *pvcsp* (VK210) present in patients with vivax malaria in Honduras is not possible to establish a hypothesis regarding the relationships between vector species and genotypes of *P. vivax*.

A phylogenetic analysis was done comparing the 84 sequences from Honduras and 9 sequences obtained from parasites collected in Latin America and Asia (Figure 3). The sequences clustered into two divisions. Division A consisted of three subdivisions that grouped 63 Honduran isolates and included one Colombian sequence (GenBank: GU339059). Division B clustered the remaining 21 Honduran sequences and three Brazilian isolates (GenBank: DQ978676; M11926; FJ845390). Sequences from Chiapas, Mexico (GenBank: JN689931) and Asia (GenBank: Iran AY632300; China S73385; South Korea AJ278612; and Thailand HQ011320) were located in a separate clade without representation of Honduran isolates.

The *pvmsp*-1 gene includes segments F1 to F3 [20]. Most of reports detect genetic variation in *pvmsp*-1 through differences in band size and/or a RFLP approach [20,39]. However, sequence analysis provides more information about polymorphisms within the gene. The F2 fragment includes one conserved block and two variable blocks 6 and 8. This fragment was amplified and sequenced in 51 Honduran isolates. A 1072 bp sequence was obtained (positions 2127 to 3198) and properly translated into amino acid sequences corresponding to residues 703 to 1060 of the protein. Minor band size differences during electrophoresis were observed. The number of polymorphic sites within the nucleotide sequences was 451 (42.08%).

A phylogeny tree was constructed with 51 *pvmsp*-1 sequences (Figure 3). Homologous sequences from Asia (n = 3), Oceania (n = 2), Brazil (n = 1) and monkey-adapted strains Sal-1 and Belem (n = 2) were also included. Three well-separated clusters were obtained with high bootstrap values. Clade A grouped 16 Honduran isolates together with Thailand (GenBank: GQ890873), Sal-1 (GenBank: AF435593, M75674), Indian (GenBank: AF435639), Brazilian (GenBank: AF435622) and Vanuatu (GenBank: AF435632, XM001614792) sequences. The second clade was formed by 14 sequences plus the South Korean isolate (GenBank: HP171940). A third and more populated clade contained 21 Honduran isolates exclusively, which grouped with the Belem strain. This indicates that *pvmsp*-1 sequences could be classified into three types. According to dendrograms patterns, no evident geographic correlation was revealed between Honduran isolates and those from other countries; however sequences similar to both Belem and Salvador-1 types were demonstrated in Honduras for the first time.

Similar to what was observed in *pvama*-1 and *pvcsp* dendrograms, no geographical clusters were revealed for *pvmsp-1* in Honduran isolates. This evidence may indicate that rather than being geographically delimited, *P. vivax* parasites circulating in Honduras may belong to a reproductive interchangeable population.

Synonymous (*πS*) and non-synonymous (*πN*) nucleotide diversity was estimated for the three *P. vivax* markers *pvama*-1, *pvcsp* and *pvmsp*-1. The difference between non-synonymous (dN) and synonymous (dS) mutations was found to be minor for *pvcsp* followed by *pvmsp*-1 and it was higher for *pvama*-1 (Table 2). Nucleotide diversity analysis within their sequences revealed that *pvmsp*-1 (F2 block) had the highest level of polymorphism and *pvama*-1 the lowest. Besides the gen *pvcsp* was found to differ by the number of repeats (17 to 20), the comparison of nucleotide variations within sequences included the whole fragment of 697 bp and revealed the lower percentage of polymorphic nucleotide positions (8,0%). The results support the idea that *pvmsp*-1 is under higher evolutionary pressure compared to the other two markers because of the frequent recombination processes described in that locus [40].

**Table 2:** **Nucleotide Diversity at three *Plasmodium vivax *surface antigen genes**

**Gene**		**Proportion**	**(±S.E.)**	**dN / dS**	**Polymorphic nucleotide positions (%)**
*Pvama*-1	dS	0.01537	0.00701	1.003	10.3
	dN	0.01542	0.00426		
*Pvmsp*-1	dS	0.12476	0.01392	0.863	42.08
	dN	0.10771	0.00769		
*Pvcsp*	dS	0.06714	0.01228	0.254	8.0
	dN	0.01708	0.0031		

The number of synonymous/non-synonymous differences per synonymous/non-synonymous site from averaging over all sequence pairs are shown. Standard error (S.E.) estimates are shown and were obtained by a bootstrap procedure (1000 replicates). Analyses were conducted using the Nei-Gojobori model.

*Plasmodium falciparum msp*-1 gene is divided into 17 blocks: seven highly variable, five conserved and five semi-conserved [41]. Allelic families relying on the polymorphic block 2 were determined. Twenty-three different size-based alleles were determined, representing K1 (n = 13), MAD20 (n = 5) and RO33 (n = 5). Fragment sizes ranged widely in each allelic family. The majority of these alleles were evenly represented, with frequencies below 9%. K1 family was predominant (57%) and doubled the frequency of MAD20 allelic types (25%) (Table 3).

**Table 3:** ***Plasmodium falciparum msp*-1 genotypes**

**K1 (bp)**	**n (%)**	**MAD20 (bp)**	**n (%)**	**RO33 (bp)**	**n (%)**	**Total**
149	1 (2.85)	176	3 (8.57)	205	1 (2.85)	
160	2 (5.71)	185	2 (5.71)	253	2 (5.71)	
164	1 (2.85)	200	1 (2.85)	262	1 (2.85)	
171	2 (5.71)	205	2 (5.71)	272	1 (2.85)	
188	2 (5.71)	214	1 (2.85)	285	1 (2.85)	
200	1 (2.85)					
226	1 (2.85)					
233	2 (5.71)					
239	2 (5.71)					
245	1 (2.85)					
271	1 (2.85)					
280	1 (2.85)					
290	3 (8.57)					
Total	20 (5.71)		9 (25.71)		6 (17.14)	35

Even though RO33 family has been considered less polymorphic than other alleles, amplicons were estimated to oscillate widely between 205 – 285 bp. Eight isolates revealed the presence of mixed clonal infections. Five out of eight showed specific bands for both K1 and RO33, while two showed two sizes within the K1 family. A previous published study analysing the genetic diversity of this marker in parasites collected from Honduras included samples taken between 1995 and 1996 [16]. Some discrepancies emerge when this study is compared to their results. Haddad *et al.* reported a predominance of MAD-20 alleles followed by K1 [16]. A lower genetic diversity was estimated and there was no report of RO33 alleles was made [16]. Such difference can be partly explained by the small sample collection area used (Municipality of Tocoa only) compared to the six municipalities sampled in this study (Iriona, Jutiapa, Roatán, Juticalpa, Puerto Lempira and Wampusirpe). A less suitable hypothesis points to the long period of time between the two studies. However, the prevalence of *P. falciparum* infections has sharply declined since 1996 in Honduras and a lower number of malaria cases would likely lead to a reduced genetic diversity among parasites.

Even though the sample size in this study is too small to ensure a precise determination of genetic diversity of *P. falciparum* in Honduras (n = 30), results such as high allelic diversity, predominance of K1-type sequences in *pfmsp*-1, and presence of RO33 genotypes are more similar to distribution patterns worldwide [13,42-44] than those reported in the previous publications [16].

The central region of *pfmsp-2* was amplified and sequenced with family-specific primers. According to the dimorphic nature of this domain, the gene can be grouped into two allelic families, 3D7 and FC27 [45]. Eleven isolates collected at Puerto Lempira and Juticalpa municipalities amplified a 613 bp band for *pfmsp*-2 gene. All of them belonged to the 3D7 family and only one sample also amplified for FC27 in a probable mixed clonal infection. Ten 3D7 sequences showed low variation with 606 conserved nucleotide positions. A GenBank blast search revealed a maximum similarity of 92% compared to previously reported sequences. The remaining sequence showed a 99% similarity index. The low number of samples amplifying *pfmsp*-2 could be attributed to *P. falciparum* genotypic variants present in Honduras with mutated primer annealing targets. The low similarity found with sequences deposited in the world databases may reinforce this hypothesis.

An important consideration for the correct interpretation of these results is the methodological approach chosen. Since direct sequencing of PCR products does not reveal the presence of mixed strain infections this may lead to over-represented genetic diversity. However, although cloning PCR products would be a better choice to improve the calculation of genetic diversity, this approach is time-consuming and requires a higher budget.

## Conclusion

Genetic analysis using three polymorphic markers (*pvama-1*, *pvcsp*, *pvmsp-1*) for *P. vivax* and two for *P. falciparum* (*pfmsp-1* and *pfmsp-2*) revealed a high degree of genetic diversity although Honduras is considered a low transmission area. Due to their polymorphic nature, these markers could be valuable tools to distinguish relapses, recurrences and reinfections, and to establish eventual changes in the parasite population dynamic.

## Competing interests

The authors declare that they have no competing interests.

## Authors’ contributions

ACL, AO and JC carried out the molecular work. WS assisted in the molecular work. REMT and EIB carried out the fieldwork, collected the samples and organized the microscopic diagnosis. WS and IJ were responsible for designing the study and data analysis. GAF was responsible for the study design, molecular tests implementation, and data analysis. All authors contributed to writing the manuscript.
